# Immature rat testis sustained long-term development using an integrative model

**DOI:** 10.1186/s40659-022-00398-y

**Published:** 2022-10-04

**Authors:** Yubo Ma, Juan Chen, Hecheng Li, Fangshi Xu, Tie Chong, Ziming Wang, Liandong Zhang

**Affiliations:** Department of Urology, The Second Affiliated Hospital of Xi’an Jiaotong University, No. 157 Xiwu Road, Xi’an, 710004 China

**Keywords:** Neonatal testis, Organ culture, Xenotransplantation, Spermatogenesis, Oxidative stress

## Abstract

**Background:**

Xenotransplantation has been primarily performed using fresh donor tissue to study testicular development for about 20 years, and whether the cultured tissue would be a suitable donor is unclear. In this study, we combined testicular culture and xenotransplantation into an integrative model and explored whether immature testicular tissue would survive and continue to develop in this model.

**Methods:**

In the new integrative model group, the testes of neonatal rats on postnatal day 8 (PND 8) were cultured for 4 days ex vivo and then were transplanted under the dorsal skin of castrated nude mice. The xenografted testes were resected on the 57th day after xenotransplantation and the testes of rats in the control group were harvested on PND 69. The survival state of testicular tissue was evaluated from morphological and functional perspectives including H&E staining, immunohistochemical staining of 8-OH-dG, immunofluorescence staining, TUNEL assay, ultrastructural study, gene expression and protein analysis.

**Results:**

(a) We found that complete spermatogenesis was established in the testes in the new integrative model group. Compared with the control in the same stage, the seminiferous epithelium in some tubules was a bit thinner and there were vacuoles in part of the tubules. Immunofluorescence staining revealed some ACROSIN-positive spermatids were present in seminiferous tubule of xenografted testes. TUNEL detection showed apoptotic cells and most of them were germ cells in the new integrative model group. 8-OH-dG immunohistochemistry showed strongly positive-stained in the seminiferous epithelium after xenotransplantation in comparison with the control group; (b) Compared with the control group, the expressions of FOXA3, DAZL, GFRα1, BOLL, SYCP3, CDC25A, LDHC, CREM and MKI67 in the new integrative model group were significantly elevated (*P* < 0.05), indicating that the testicular tissue was in an active differentiated and proliferative state; (c) Antioxidant gene detection showed that the expression of Nrf2, Keap1, NQO1 and SOD1 in the new integrative model group was significantly higher than those in the control group (*P* < 0.05), and DNA methyltransferase gene detection showed that the expression of DNMT3B was significantly elevated as well (*P* < 0.05).

**Conclusion:**

The new integrative model could maintain the viability of immature testicular tissue and sustain the long-term survival in vivo with complete spermatogenesis. However, testicular genes expression was altered, vacuolation and thin seminiferous epithelium were still apparent in this model, manifesting that oxidative damage may contribute to the testicular development lesion and it needs further study in order to optimize this model.

## Background

The two-dimensional models such as cell culture are important method to study testicular development and spermatogenesis but cannot effectively mimic the complex architecture of the seminiferous tubule. As growing numbers of studies indicates the importance of extracellular matrix for tissue development and function in recent years [1–3], the development and optimization of a variety of in vivo and in vitro models including testicular tissue culture and transplantation has achieved a transformation from two- to three-dimension. Spermatogonial stem cells (SSCs) that play important role in maintaining continuous spermatogenesis throughout the male reproductive lifespan are characterized by the ability to self-renewal, division, and differentiation [4]. Previous studies have demonstrated that Leydig cells, macrophages, vasculature-associated cells, peritubular cells, and paracrine products of those cells are necessary for normal SSCs activity [5]. Thus, the main advantage of using testicular tissue to study spermatogenesis is the presence of those components that can maintain the testicular niche structure and mimic the testicular microenvironment [6].

In fact, various studies using testis tissue culture and transplantation were done to make clear testicular development and the effects after endocrine-disrupting chemicals (EDCs) exposure. Sato obtained spermatids and sperm by positioning testicular fragments of neonatal mouse at the gas–liquid interphase with application of knock-out serum replacement (KSR) in media [7]. Zhang et al.evaluated the effects of multiple EDCs exposure at a low dose on fetal testis by using this organ culture system, but the long-term effects were not observed because of the limitation of culturing duration [8]. Honaramooz et al.reported the establishment of complete spermatogenesis after grafting neonatal testis tissue into mouse hosts in 2002 [9]. Fayomi et al.observed complete spermatogenesis by autologously grafting testicular tissues under the back skin and scrotal skin of castrated pubertal rhesus macaques, and the graft-derived sperm were confirmed to give birth to a healthy female baby after fertilization [10]. Gassei et al.xenografted Matrigel patches that contained testicular cordlike structures after 10 days of in vitro culture and those aggregates transplanted under the back skin of nude mice grew into larger, but intact testicular structure was not observed [11]. With the optimization of organ culture system, testis organ culture has been widely used in the field of andrology especially the investigation of effects of chemicals alone or in mixture upon the testis development and function. The advantages of testis organ culture model include (a) the precise control of the duration and level of EDCs exposure; (b) reducing the number of experimental animals and minimizing individual differences [12]. However, it also has inherent limits including (a) the short duration of culture; (b) failing to study the potential testicular feedback loops and the indirect effects that chemicals can exert [12]. By contrast, testicular tissue transplantation shows some strengths that could compensate those limits including (a) a long survival duration of testicular tissue in vivo; (b) establishing a hormonal interaction between hypothalamus and pituitary of the host and the grafted tissue [13]. Consequently, using organ culture system could observe EDCs effects on spermatogenesis in short term, while tissue transplantation could provide a more suitable environment for observing the long-term effect that chemicals can exert such as EDCs. Then, a combination of organ culture and transplantation might be a positive method for related studies.

At present, testicular culture and transplantation are commonly used in fertility preservation and gonadotoxicity research of EDCs on testicular function [14, 15], however, few experiments have explored the successive development of immature testes after short-term culture ex vivo, indicating the results we obtained were of great significance. In this study, we established integrative model of immature neonatal testicular tissue using 8 days-old rats and evaluated the survival state of xenografted testes from morphological and functional perspectives, hoping to provide insight into fertility restoration strategies and the immature testis developmental pattern under the influence of chemicals in different species.

## Materials and methods

### Sample collection

Specific pathogen free (SPF) experimental animals including pregnant female Sprague–Dawley (SD) rats and BALB/c male nude mice were obtained from the Experimental Animal Center of Xi’an Jiaotong University. Six sets of models from six different litter were performed and each litter of neonatal rats was randomly divided into control and experimental group. Part of the male offspring was executed on postnatal day 8 (PND 8) and the testes were removed aseptically and then immediately transferred into a culture medium on ice for later culture. The other were fed normally until PND 69 as control.

### Organ culture protocol

**Fig. 1:**
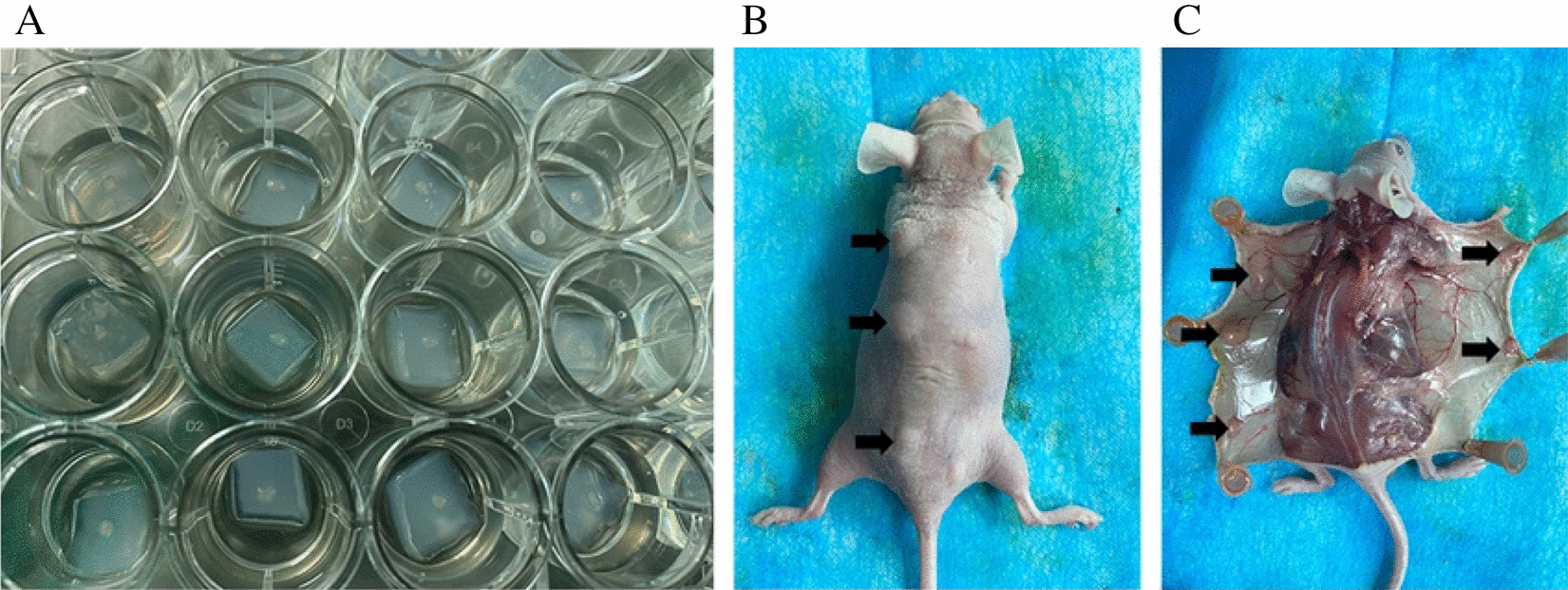
The organ culture system (**A**) and the testes (↑) after xenotransplantation for 57 days (**B**, **C**)

Before organ culture, a 5 mm thick agarose gel formed by 1.5% hot agarose solution composed of agarose powder and double-distilled water was prepared. The next step was to cut agarose gel into 10 ×5 ×5mm^3^ pieces with a sterile blade and then soak those pieces in culture medium overnight. After sliced into small pieces (1–2 mm^3^) on ice, the isolated neonatal testes were transferred onto the surface of agarose gel half-soaked in the 24-well plate in which 600 μL of culture medium was added (Fig. 1A). The culture medium was DMEM/F12 (cat# 11039-021, Gibco, USA) supplemented with 10% KSR (cat# A31815-01, Gibco, USA), penicillin (100 IU/mL) and streptomycin (100 μg/mL) (cat# P1400, Solarbio, China). One piece of testis was held on each gel brace, and a medium was changed every 2 days. The testes were incubated at 37 °C in a humidified incubator containing 5% CO_2_ and 95% air for 4 days. Part of the tissues was used for xenografting 4 days after culture and the rest was fixed for histology.

### Xenografting

Six BALB/c male nude mice aged 4 weeks were acclimated for 3 days in experimental animal center and then were castrated under anesthesia and the testicular tissue that had been cultured for 4 days was transplanted under the dorsal skin of recipient nude mice 2 weeks after castration. Three grafts per rat were transplanted on either side of the midline. Keeping warm after surgery of castration and xenotransplantation and giving antibiotics in drinking water for 3 days were necessary. Experimental animals kept in the experimental animal center were treated with prepared water and food under 12 h light/dark cycle.

The xenografted testes on the dorsal skin of recipient were resected on the 57th day after xenotransplantation (Fig. 1B–C). For the control group, the testes of male rats were harvested on the same day, namely PND 69. The collected testes were divided into two parts, one was used to be fixed for histology and ultrastructural study, another was used for gene and protein analysis.

### Testicular histology

For fixation, testicular tissue was immersed in 4% paraformaldehyde fixative solution at 4 °C for 6 h. After dehydration in ethanol and xylene, the samples were embedded in paraffin and cut into 5 μm sections. At last, sample staining was done with hematoxylin for 1–2 min and eosin for 1 min. Diameter of seminiferous tubules was measured by using Image J version 1.8.0 (National Institutes of Health, USA). Fifty tubule cross sections of testicular tissue before and after culture, xenografted testes and control were randomly selected in each setting. Observation and evaluation under the microscope were performed by an independent investigator.

### Immunohistochemistry

Immunohistochemical staining used the sections made above. Sections were deparaffinized in xylene and rehydrated in graded ethanol series. After antigen retrieval with sodium citrate buffer (cat# C02-02002, Bioss, China) at 98 °C for 10 min and endogenous peroxidase blocking with normal goat serum, the sections were incubated in a humidified box at 4 °C overnight with rabbit anti-8-OH-dG polyclonal antibody (1:500, cat# bs-1278R, Bioss, China). The secondary antibody was biotin-labeled goat anti-rabbit IgG (cat# SP-0023, Bioss, China), and 3,3′-diaminobenzidine (DAB, cat# C-0010, Bioss, China) staining was performed under the microscope. Diluting solution was used to replace primary antibody for the negative control.

### Immunofluorescence

After deparaffinization, rehydration and antigen retrieval, sections were incubated with Triton X-100 for 10 min and blocking buffer (cat# P0102, Beyotime, China) for 1 h at room temperature. Primary antibody (ACROSIN, 1:100, cat# NBP2-14260, Novus Biologicals, USA) was added to tissue sections at 4 °C for a night, followed by incubating with secondary antibody (1:1000, cat# P0176, Beyotime, China) without light at room temperature for 1 h. After counterstaining with DAPI (cat# C1005, Beyotime, China), sections were observed under fluorescence microscope. The seminiferous tubules were randomly selected for each group and the number of spermatids in the ACROSIN-positive tubules was counted and compared between the control and new integrative model groups.

### Apoptosis assay

A TUNEL apoptosis assay kit (cat# C1098, Beyotime, China) was used to detect apoptosis according to the manufacturer’s instructions. After deparaffinized and hydrated, the sections were incubated with 20 μg/mL DNase-free proteinase K at 37 °C for 20 min, followed by washing with PBS for 3 times and blocking endogenous peroxidase with 3% H2O2 in PBS at 25 °C for 20 min. Subsequently, the sections were incubated with working solution containing biotin-dUTP and TdT enzyme at 37 °C away from light for 60 min. Then the sections were washed again and incubated with streptavidin–HRP solution, followed by DAB solution for color developing. Two or more TUNEL-positive cells existed in a seminiferous tubule was considered as positive. The ratio of number of apoptotic positive tubules and total number of tubules in a cross section was counted as apoptosis index (AI).

### Ultrastructural study

Washing tissue promptly with PBS after harvested and then immersing in 4% formaldehyde and 2.5% glutaraldehyde in PBS at 4 °C for 2 h was the first step, followed by post-fixed in 1% osmium tetroxide at 4 °C for 2 h. Subsequently, the sections were double stained with uranyl acetate for 15 min and lead citrate for 5 min after dehydrating, embedding, and sectioning. Observation under H-7650 transmission electron microscope (TEM) at 80 kV (Hitachi, Japan) was done by an independent researcher.

### Real-time quantitative PCR

The detailed procedure of RNA extraction was based on the protocol of TaKaRa MiniBEST Universal RNA Extraction Kit (cat# 9767, Takara, Japan), and the PrimeScript^™^ RT Master Mix (cat# RR036A, Takara, Japan) was used to synthesize cDNA. Real-time quantitative PCR was done using TB Green Premix Ex Taq II (cat# RR820A, Takara, Japan) with total reaction volume of 20 μl on the Bio-Rad CFX Connect Real-Time PCR Detection System (Bio-Rad, USA). GAPDH was assayed in all samples as an internal control. The amplification process was initiated with a denaturation cycle at 95 °C for 2 min, followed by 39 cycles of 95 °C for 10 s and 60 °C for 30 s. All analyses were performed in triplicate samples. Quantitative analysis of gene expression was evaluated by the method of 2^−ΔCt^ algorithm. The gene names and primer sequences are listed in Table 1.

**Table 1:** The genes and primer sequences

Gene name	Accession No	Forward primer	Reverse primer
AMH	NM_012902.1	5-CTAACCCTTCAACCAAGCAAAG-3	5-GGAGTCATCCGCGTGAAA-3
BOLL	NM_001113370.1	5-AACAGCCTGCATATCACTACC-3	5-GCAGATATAGGAATGGAGCAGAA-3
CDC25A	NM_133571.1	5-GTGAACTTGCACATGGAAGAAG-3	5-CTCACAGTGGAACACGACAA-3
CDKN1A	NM_080782.4	5-CCTAAGCGTACCGTCCAGAG-3	5-GAGAGCAGCAGATCACCAGATTA-3
CDKN1B	NM_031762.3	5-GATGTAGTGTCCTTTCGGTGAGA-3	5-ACTCCCTGTGGCGATTATTCAA-3
CREM	NM_001110860.2	5-GCCAGGTTGTTGTTCAAGATG-3	5-TGTGGCAAAGCAGTAGTAGG-3
CYP11A1	NM_017286.3	5-AGAACATCCAGGCCAACATC-3	5-CCTTCAAGTTGTGTGCCATTTC-3
DAZL	NM_001109414.1	5-AGTCCAAATGCTGAGACATACA-3	5-TGAACTGGTGAACTCGGATAAG-3
DNMT1	NM_053354.3	5-ACTTTCTCGAGGCCTACAATTC-3	5-TTTCCCTTCCCTTTCCCTTTC-3
DNMT3A	NM_001003958.1	5-CCACCAGGTCAAACTCCATAAA-3	5-GCCAAACACCCTTTCCATTTC-3
DNMT3B	NM_001003959.1	5-CGACAACCGTCCATTCTTCT-3	5-GTCGATCATCACTGGGTTACAT-3
FOXA3	NM_017077.2	5-GCTGACCCTGAGTGAAATCTAC-3	5-TCATTGAAGGACAGCGAGTG-3
FSHR	NM_199237.1	5-TGTGCCAATCCTTTCCTCTAC-3	5-TGTAAATCTGGGCTTGCATTTC-3
GAPDH	NM_017008.3	5-GGCACAGTCAAGGCTGAGAATG-3	5-ATGGTGGTGAAGACGCCAGTA-3
GFRα1	NM_012959.1	5-GTGCTCCTATGAAGAACGAGAG-3	5-TGGCTGGCAGTTGGTAAA-3
HO-1	NM_012580.2	5-GTCCCTCACAGACAGAGTTTC-3	5-AACTAGTGCTGATCTGGGATTT-3
HSD3B3	NM_001042619.1	5-TTCCTGCTGCGTCCATTT-3	5-GATCTCTCTGAGCTTTCTTGTAGG-3
INHBB	NM_080771.1	5-CGAAGGCAACCAGAACCTATT-3	5-TACACCTTGACCCGTACCTT-3
Keap1	NM_057152.2	TCCTCAGAGGGCAGTGGAAT	TATGTGTCCCACAAGGGAGC
LDHC	NM_017266.2	5-ATAGGATCCGACTCCGATAAGG-3	5-GCAATGGCCCAAGAGGTATAG-3
MKI67	NM_001271366.1	5-CCGTAGAATTGGCTGGTCTCA-3	5-AGGCTATCAACTTGCTCTGGTT-3
Nrf2	NM_031789.2	5-ACGTGATGAGGATGGGAAAC-3	5-TATCTGGCTTCTTGCTCTTGG-3
NQO1	NM_017000.3	5-GCTGCAGACCTGGTGATATT-3	5-ACATGGTGGCATACGTGTAG-3
PHB	NM_031851.2	5-CATCACACTACGTATCCTCTTCC-3	5-CTTGAGGATCTCTGTGGTGATAG-3
SHBG	NM_012650.1	5-AAGGACAGAGACTGGACATAGA-3	5-TTAGTGGGAGGTGTGGGTAT-3
SOD1	NM_017050.1	5-GGTCCACGAGAAACAAGATGA-3	5-CAATCCCAATCACACCACAAG-3
SOD2	NM_017051.2	5-AGCGTGACTTTGGGTCTTT-3	5-AGCGACCTTGCTCCTTATTG-3
SOD3	NM_012880.1	5-GAGATCTGGATGGAGCTAGGA-3	5-ACCAAGCCTGTGATCTGTG-3
SYCP3	NM_013041.1	5-GAGCCAGAGAATGAAAGCAATC-3	5-GTTCACTTTGTGTGCCAGTAAA-3
TSPO	NM_012515.2	5-CTATGGTTCCCTTGGGTCTCTA-3	5-AAGCATGAGGTCCACCAAAG-3
WT-1	NM_031534.2	5-CACCAGGACTCATACAGGTAAA-3	5-TGTTGTGATGGCGGACTAA-3

### Western blotting

The total proteins in the testicular tissue were extracted by using lysis buffer (cat# P0013C, Beyotime, China). Three different samples were performed in each group. Then the protein lysates were separated by 8–12% SDS-PAGE (25 min: 90 V, and 100 min: 120 V, room temperature), and electro-transferred (90 min: 200 mA, 4 °C) onto the polyvinylidene fluoride membranes. After incubating in blocking buffer for 2 h, the membranes were incubated overnight in primary antibodies (Nrf2, 1:1250, cat# 16396-1-AP, Proteintech, USA; Keap1, 1:2500, cat# 10503-2-AP, Proteintech, USA; NQO1, 1:5000, cat# ab80588, Abcam, UK; SOD1, 1:5000, cat# ab51254, Abcam, UK; GAPDH, 1:2500, cat# AF1186, Beyotime, China) at 4 °C, and then incubated for 2 h in HRP-conjugated secondary antibodies (1:2000, cat# SA00001-2, Proteintech, USA). The final detection was performed by using ECL reagent (cat# P0018FS, Beyotime, China). Image gray value was analyzed using Image J version 1.8.0.

### Statistical analysis

Data were expressed as mean ± SEM. Significant differences were identified by unpaired two-tailed *t*-test or Welch’s *t*-test using GraphPad Prism version 8.0 (GraphPad, USA). Differences were considered as significant at *P* < 0.05.

## Results

### Testicular histology

**Fig. 2:**
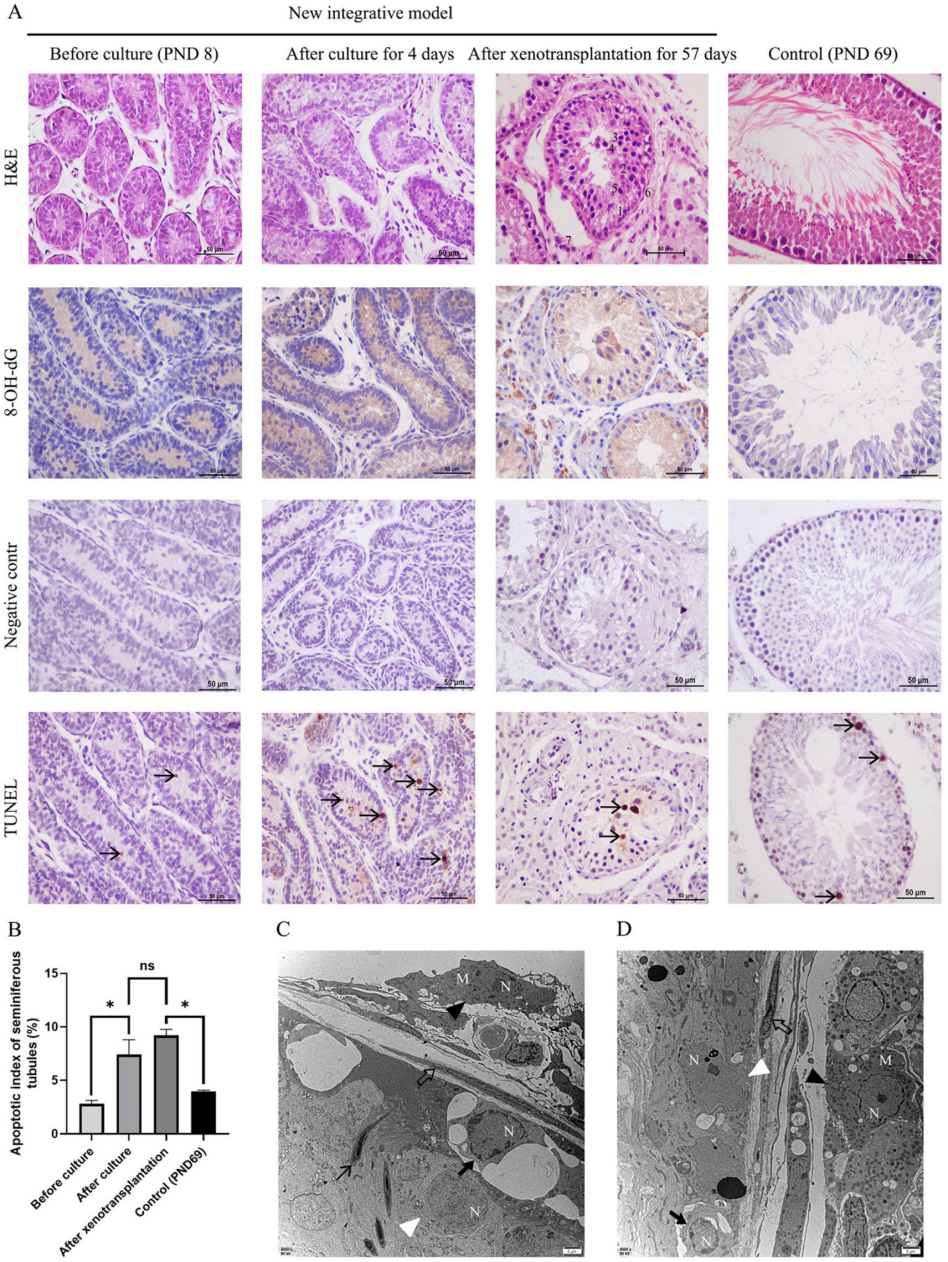
**A**: **H**&**E** staining, immunohistochemical staining of 8-OH-dG and TUNEL assay of rat testis in the control and new integrative model groups. **B**: The results showed that there were more apoptotic cells (↑) after culture than that before culture and more apoptotic cells in the new model group than that in the control group. **C**: Ultrastructural study of rat testis in the control group. **D**: Ultrastructural study of rat testis in the new integrative model group. (1: spermatogonium; 2: primary spermatocyte; 3: secondary spermatocyte; 4: spermatid; 5: Sertoli cell; 6: myoid cell; 7: Leydig cell; Black filled triangle: Leydig cell; white filled triangle: Sertoli cell; white filled arrow: myoid cell; ↑: elongating spermatid; black filled arrow: spermatocyte; N: nuclei; M: mitochondria; *Significant difference at P < 0.05; *ns* no significant difference; **A**: scale bars indicate 50 µm; **C**, **D**: scale bars indicate 2 µm.)

**Fig. 3:**
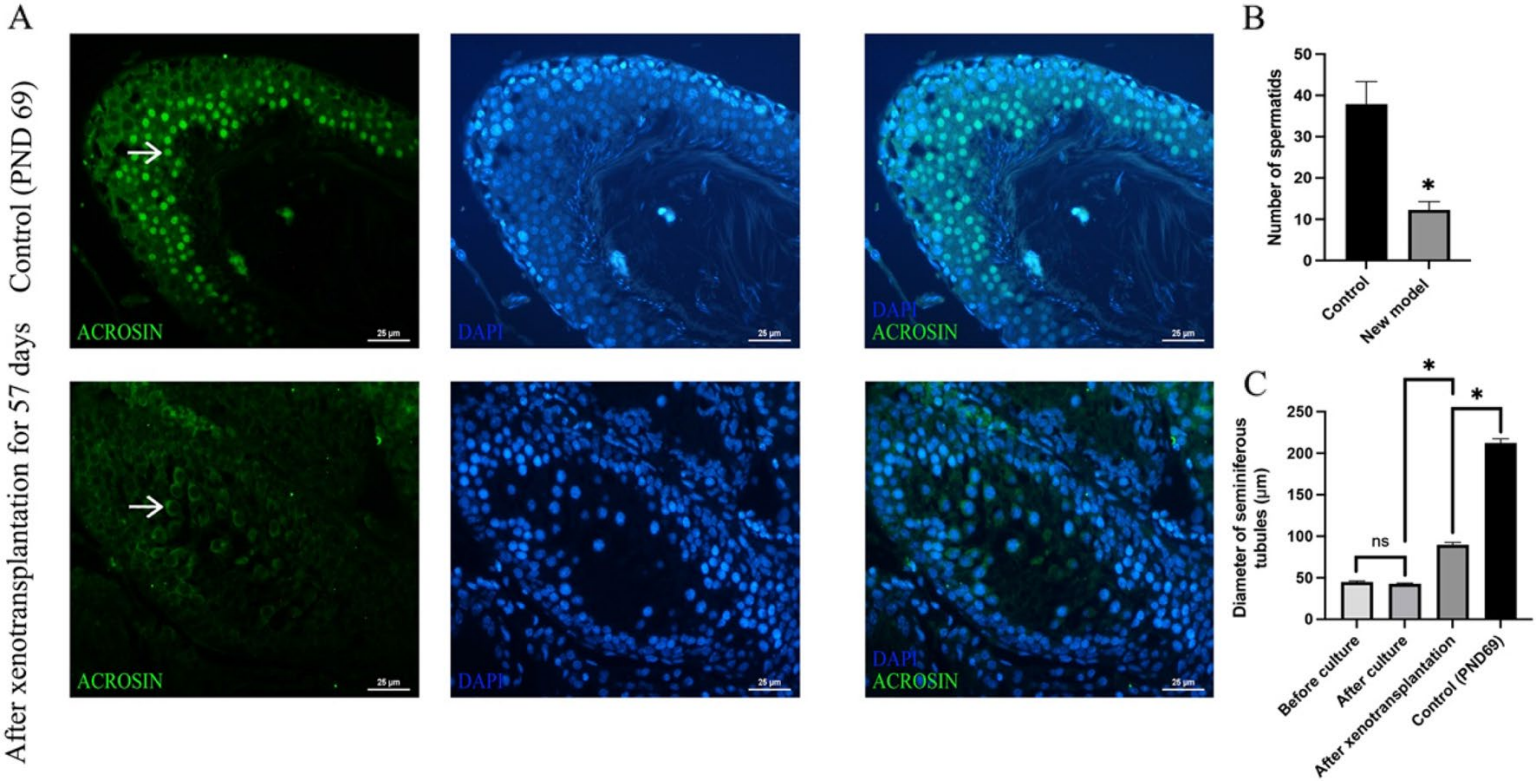
**A**: Immunofluorescent analyses of rat testis in the control and new integrative model groups. **B**: In the ACROSIN-positive tubules, spermatids in the control was significantly more than in the new model group. **C**: Diameter of seminiferous tubules in different settings. (white filled arrow:Spermatid; *Significant difference at P < 0.05; *ns* no significant difference; scale bars indicate 25 µm.)

H&E of testicular sections are shown in Fig. 2A. Before culture, H&E staining showed intact testicular structure without necrosis or vacuoles. Seminiferous tubules were well established and spermatogonia were seen close to the basement membrane. After cultured for 4 days, the lumen was gradually enlarged within intact seminiferous tubules but the diameter of tubules was not significantly changed in statistics (42.79 ± 0.93 versus 44.92 ± 1.27 μm, *P* > 0.05, Fig. 3C). After xenotransplantation for 57 days in nude mice, H&E staining showed intact testicular structure with larger diameter (89.59 ± 2.99 μm) of the seminiferous tubules in comparison with the cultured testes (*P* < 0.05, Fig. 3C); spermatogonia were close to the basement membrane with round nuclei; primary spermatocytes were round in large volume and the cells in different stages of meiosis could be seen; secondary spermatocytes were close to the proximal lumen with dark nuclei; spermatids were close to the proximal lumen with small volume; Sertoli cells were irregularly tapered with triangular or oval nuclei; myoid cell were near the basement membrane in spindle shape; Leydig cells were located between the seminiferous tubules with round nuclei. Moreover, compared with the control group (212.30 ± 5.08 μm) in the same stage, the diameter of seminiferous tubules was significantly less (*P* < 0.05, Fig. 3C) and there were vacuoles and thinner seminiferous epithelium in part of the tubules, indicating that the testis development and testicular cell differentiation was disrupted in this integrative model.

### Immunohistochemistry

To observe DNA oxidative damage in xenografted testes, 8-OH-dG was detected using immunohistochemistry on paraffin sections (Fig. 2A). 8-OH-dG was lightly positive-stained in the lumen before and after culture. While the testicular tissue was strongly positive-stained in the seminiferous epithelium and interstitial cells after xenotransplantation in comparison with the control group, which indicated that the neonatal rat testis culture and xenotransplantation might inevitably aggravate DNA oxidative damage.

### Comparison of TUNEL assay

The TUNEL assay is shown in Fig. 2A. The rate of TUNEL-positive cells was generally very low before culture, while a little more TUNEL-positive germ cells were observed after cultured for 4 days and the main cell type was spermatogonium. As for the tissue after xenotransplantation, some TUNEL-positive germ cells fell out from the seminiferous epithelium while there were a few positive cells in the control group. AI value in the testes after culture were statistically higher than that before culture (*P* < 0.05) and AI value in the new model group were statistically higher than that in the control group (*P* < 0.05) as well, while there was no difference between the testes after culture and after xenotransplantation (Fig. 2B), indicating that oxidative stress may play a role in germ cell apoptosis in seminiferous epithelium and xenotransplantation in this new integrative model group might have no ability to promote apoptosis.

### Ultrastructural study

The ultrastructure of testis is shown in Fig. 2C–D. In those two groups, seminiferous tubules were surrounded by intact basement membrane and myoid cells. The seminiferous epithelium in the control group was well arranged, which consists of spermatocytes, spermatids and Sertoli cells. Sertoli cells were identified by their round but bigger nucleus with weaker electron density. While in the integrative model group, the spermatids were rarely seen. Both groups had abundant mitochondria in Leydig cells and no obvious swelling was observed in mitochondria and endoplasmic reticula.

### Immunofluorescence

To observe the spermatids in the control and new integrative model groups, ACROSIN was used to mark spermatids (Fig. 3A). Immunofluorescence staining revealed that some ACROSIN-positive spermatids (green) were present in seminiferous tubule cross sections of xenografted testes, while the more spermatids in the control under the same magnification. Merged images showed that spermatids would be closed to the lumen, which was consistent with H&E staining. In the ACROSIN-positive tubules, there were significantly more spermatids in the control (37.88 ± 5.46) than in the new integrative model group (12.25 ± 2.00, *P* < 0.05, Fig. 3B).

### Gene expression of Sertoli cell markers

**Fig. 4:**
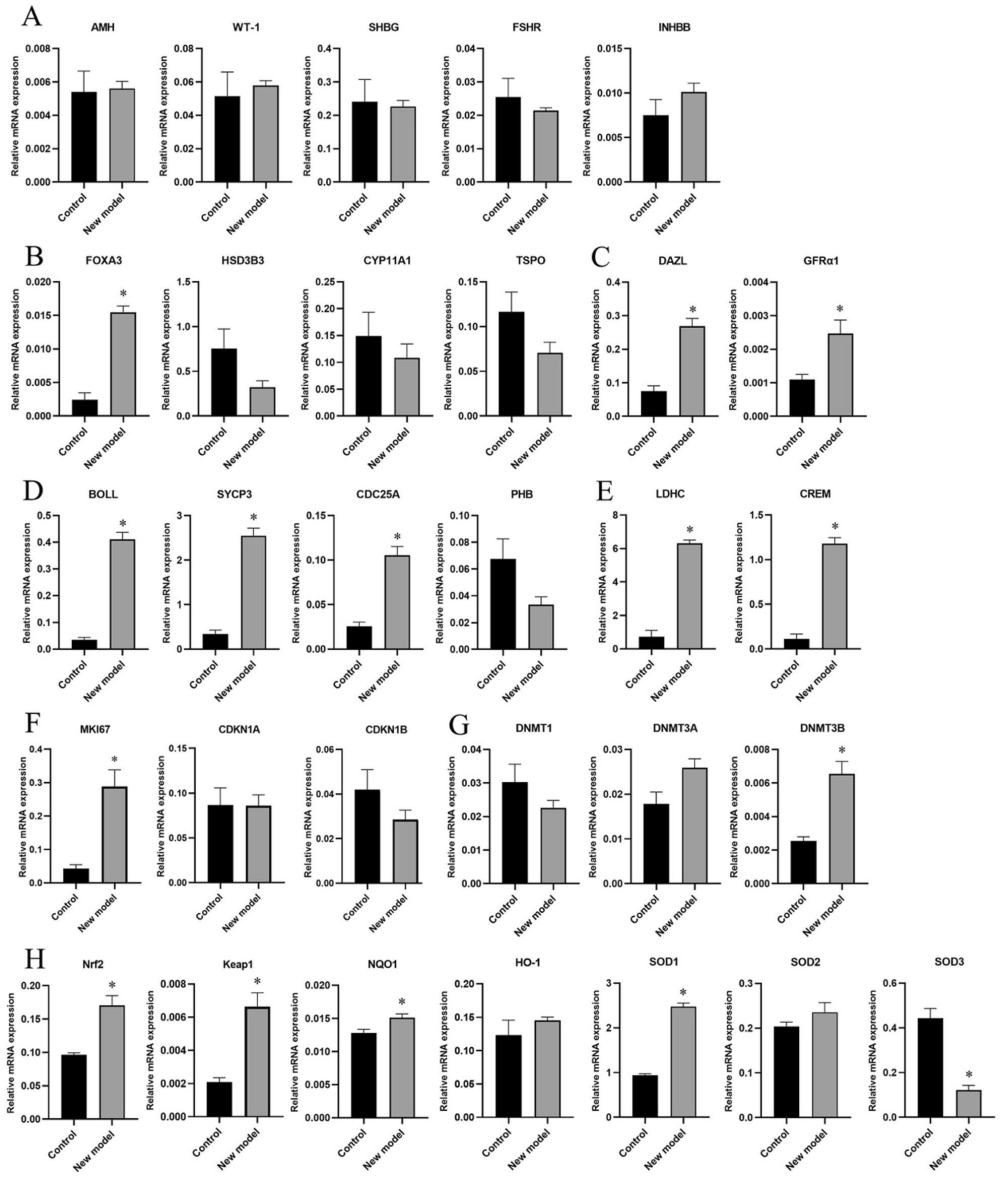
Gene expression in different cell markers and antioxidative pathway in the control and new integrative model groups. **A**: Sertoli cell; **B**: Leydig cell; **C**: mitotic germ cell; **D**: meiotic germ cell; **E**: spermiogenesis; **F**: cell proliferation; **G**: DNA methyltransferase; **H**: antioxidative pathway; *Significantly different from control at P < 0.05.)

The gene expression of Sertoli cell markers of each group is shown in Fig. 4A. No significant difference was found in AMH, WT-1, SHBG, FSHR, and INHBB expression between the two groups (*P* > 0.05).

### Gene expression of Leydig cell markers

The gene expression of Leydig cell markers is shown in Fig. 4B. The expression of FOXA3 in the new model group was significantly higher than that in the control group (*P* < 0.05). No significant difference was found in HSD3B3, CYP11A1, and TSPO expression between the two groups (*P* > 0.05).

### Gene expression of mitotic germ cell markers

The expression of mitotic germ cell markers is shown in Fig. 4C. The expression of DAZL and GFRα1 in the new model group was significantly higher than that in the control group (*P* < 0.05).

### Gene expression of meiotic germ cell and spermiogenesis markers

The expression of meiotic germ cell markers is shown in Fig. 4D. The expression of BOLL, SYCP3, CDC25A in the new model group was significantly higher than that in the control group (*P* < 0.05). There was no significant difference in PHB expression between the two groups (*P* > 0.05). In terms of the spermiogenesis markers (Fig. 4E), LDHC and CREM expressions in the new model group was significantly higher than that in the control group (*P* < 0.05).

### Gene expression of cell proliferation markers

The gene expression of cell proliferation markers is shown in Fig. 4F. The expression of MKI67 in the new model group was significantly higher than that in the control group (*P* < 0.05). No significant difference was observed in CDKN1A and CDKN1B expression between the two groups (*P* > 0.05).

### Gene expression of DNA methyltransferase

The gene expression of DNA methyltransferase is shown in Fig. 4G. The expression of DNMT3B in the new model group was significantly higher than that in the control group (*P* < 0.05). No significant difference was found in DNMT1 and DNMT3A expression between the two groups (*P* > 0.05).

### Gene expression of antioxidative genes

The expression of antioxidative genes is shown in Fig. 4H. The expression of Nrf2, Keap1, NQO1 and SOD1 in the new model group was significantly higher than that in the control group (*P* < 0.05), while SOD3 was significantly lower than that in the control group (*P* < 0.05). No significant difference was found in HO-1 and SOD2 expression between the two groups (*P* > 0.05).

### Protein analysis of Keap1-Nrf2-ARE signaling pathway

**Fig. 5:**
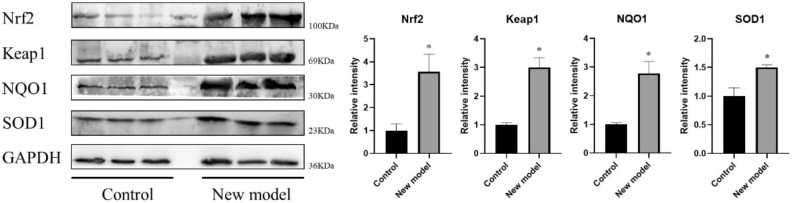
Western blot of related genes in Keap1-Nrf2-ARE signaling pathway. (Three different samples in each group; *Significantly different from control at P < 0.05.)

In accordance with alternations of mRNA expression, western blot showed the same trend (Fig. 5). The relative intensity of Nrf2, Keap1, NQO1 and SOD1 in the new model group was significantly higher than that in the control group (*P* < 0.05).

## Discussion

Xenografting has been mainly performed using fresh or cryopreserved donor tissue in different species [16, 17], but cultured testicular tissues we used showed comparable graft survival as well. Although the percentage of seminiferous tubules with differentiated germ cells appeared to increase after refrigeration [18], optimization and adaptation of current protocols was necessary due to species-specific differences in order to define suitable conditions.

The purpose of this research was to explore the viability of xenografted testes which were cultured ex vivo for 4 days before grafting. In our study, we established a new integrative model of immature neonatal testicular tissue and adopted organ culture system at first stage, which could not only simulate the physiological environment of testis in vivo, but also minimize individual differences as one testis could be sliced into several pieces. However, the cultured testes cannot be maintained for long duration because testicular structure distortion and vacuoles occurred apparently with time extending. At the second stage of this integrative model, we successfully xenografted the cultured tissue of testes which could continue to develop under the dorsal skin of nude mice for approximately 8 weeks.

In the testicular histology, H&E staining showed complete spermatogenesis in xenografted testes while less spermatids were found in comparison with the control group and the vacuoles and thin seminiferous epithelium occurred in part of the tubules in xenografted testes as well. Sertoli cells have supporting and nutritive functions on spermatogenic cells and participate in the formation of blood-testis barrier, which contributes to maintain a stable environment for spermatogenesis. The vacuolation of Sertoli cells is regarded as an early characteristic of morphological injury, prior to germ cell degeneration [19]. For the vacuoles in xenografted testes usually existed between or within Sertoli cells, the emergence of vacuolation is indicative of a breakdown in Sertoli–germ cell junctions and degeneration of germ cells [20]. More importantly, the oxidative injuries resulted from oxidation and antioxidant imbalance would also play a role in the formation of vacuoles and thin seminiferous epithelium. Ma reported the phenomenon of apparent vacuoles and oxidative damages after xenotransplantation of prepubertal rat testicular tissue [21]. Even though some spermatids could be identified in the xenografted testes under fluorescence microscope, there was few spermatids could be observed directly under TEM, which would be related to the vacuolation of Sertoli cells and location of sampling. ACROSIN is a serine protease found in the spermatids, and is always used as markers for spermatids [7, 10, 22]. Consequently, we used ACROSIN-positive staining to identify spermatids. TUNEL assay showed that apoptotic cells in xenografted testes were mainly germ cells, but most of the seminiferous tubules were in normal morphology without apparent necrosis. The immunohistochemistry of 8-OH-dG showed the DNA oxidative damage of xenografted testes became aggravated in comparison with the control group, indicating the xenografted testes in this integrative model could continue to develop for a period in nude mice but accompanied with oxidative damage at the meantime.

In order to evaluate the level of gene expression in different cell markers and antioxidative pathway in xenografted testes, we performed real-time PCR detection. We found that there was no significant difference in AHM, WT-1, SHBG, FSHR and INHBB expression between the two groups, indicating Sertoli cells in xenografted testes could maintain the similar physiological state to that in situ. Sertoli cells are essential for normal spermatogenesis because of their function on nutrient supply, maintenance of cell junctions, and support for germ cells mitosis and meiosis by regulation of the MAPK, AMPK, and TGF-β/Smad signaling pathways during spermatogenesis [23]. AMH, a glycoprotein produced by Sertoli cells in fetal and postnatal testes, plays an essential role in sexual differentiation and gonadal function and serum AMH serves as a good marker for Sertoli cell development in mammals [24]. WT-1 is exclusively expressed by Sertoli cells in testes and Rao found that the WT-1 gene suppression led to decreased number of sperm [25]. In this study, we used castrated nude mice as recipients, which could avoid the host testis interfering with xenografts responding to host gonadotropins. Moreover, a feedback axis would be re-established between the hypothalamus and pituitary of the host mouse and the xenografted tissue, which provided endocrine basis for spermatogenesis [13]. FOXA3 is mainly expressed in Leydig cells and plays a role in maintaining the balance of testicular testosterone by fine-tuning the expression of steroidogenic genes [26]. And we found the expression of FOXA3 was significantly higher in the new integrative model group while other genes about steroidogenesis were not, which indicated the function of Leydig cells was less affected. Spermatogenesis is a highly orchestrated developmental process that involves three parts: mitosis, meiosis and spermiogenesis. And more than half of the markers related to spermatogenesis in this study were significantly up-regulated. DAZL is mainly expressed in the early stage of spermatogenesis, which promotes proliferation and differentiation of progenitor spermatogonia by heightening the translation of thousands of genes [27] and GFRα1 is a conserved marker that appears to be most restricted to undifferentiated spermatogonia, which is used to isolate and enrich undifferentiated spermatogonia [28]. BOLL, an RNA binding protein, is an evolutionarily conserved member of the deleted in azoospermia gene family, which could cause spermatogenic arrest and sperm maturation failure in many species if lack of expression [29]. CDC25A is one of the regulator genes of meiotic progression, and decreased expression of CDC25A is associated with failure of spermatogenesis and sperm retrieval [30]. The other upregulated genes were involved in synaptonemal complexes formation (SYCP3), transcriptional regulation (CREM) and sperm motility maintenance (LDHC), finally leading to complete spermatogenesis as indicated in the histological findings. SYCP3 is a DNA-binding protein and a structural component of the synaptonemal complex and it is considered to mediate the synapsis or homologous pairing of chromosomes [31]. LDHC is testis-specific and plays a vital role for sperm motility by facilitating the conversion of L-lactate and nicotinamide adenine dinucleotide to pyruvate and the reduced form of NADPH [32]. CREM is a cAMP-related transcription factor, which plays a role in late round spermatid development and initiation of elongation [33].

In summary, we found elevated expression of mitotic, meiotic and post-meiotic markers as well as Leydig cell marker FOXA3 in our study. And in another study, elevated HSD3B and LHRH expression was found in the grafted rat testis at 8-week post-grafting [34]. It has been demonstrated that following castration FSH and LH secretion increased markedly [35] due to the loss of testicular hormones in the host. After establishment of blood supply with host, elevated levels of FSH and LH have been suggested to stimulate Sertoli cells proliferation and support Leydig cell maturation, which would support the grafted testis development and spermatogenesis. Previous studies also observed complete spermatogenesis was established from testicular cords containing only gonocytes and immature Sertoli cells at the time of grafting in part of donor species [9, 36]. After xenotransplantation, the immature rat testis was immediately exposed to a fully active hypothalamic-pituitary axis and the time to puberty appears to be accelerated in the xenografts [37]. Collectively, the shortened quiescent period of the hypothalamic-pituitary–gonadal axis and the support of the host’s endocrine system [15] contributed to grafted testis maturation and spermatogenesis establishment. Although the seminiferous epithelium in some tubules were a bit thinner and vacuoles in part of the tubules were observed, the elevated gene expression indicated that testicular tissue was in an active differentiated and proliferative state, and the immunofluorescence staining of ACROSIN-positive spermatids in the xenografted testes further confirmed that.

Spermatogenesis is a high energy-demanding process and low levels of oxidative stress are essential for normal testicular function. Testes are equipped with a potent antioxidant system in the physiological state in order to resist oxidative injuries [38]. Immunohistochemistry in this study showed strongly positive 8-OH-dG in seminiferous epithelium after xenotransplantation, and more TUNEL-positive germ cells were observed after culture, indicating that new integrative model (culture and xenotransplantation) of immature neonatal rat testes inevitably disrupted normal spermatogenesis lightly accompanied with aggravated oxidative damage. The Keap1-Nrf2-ARE signaling pathway performs a critical role in maintaining the cellular redox balance and metabolism and inducing an adaptive response for oxidative stress [39]. And Nrf2 would be liberated from the Keap1-Nrf2 complex under conditions of oxidative stress [40]. The target genes of Nrf2 include NQO1, SOD, HO-1 and so on. Considering the ischemia and hypoxia are inevitable until a functional circulatory connection is established between host and grafts, and in our study, we found Nrf2, Keap1, NQO1, SOD1 were significantly upregulated in comparison with the control group, which suggested that the grafted testes reacted to the oxidative stress and increased the expression of Nrf2 and targets including NQO1 and SOD1 in order to reduce oxidative damage. Moreover, the imbalance of oxidation and antioxidant also had impact on the morphology of xenografted testes, inevitably leading to the formation of vacuoles and thin seminiferous epithelium.

In addition to cell differentiation, proliferation is throughout testis development as well. MKI67, a nuclear protein expressed in all proliferating vertebrate cells, is present during all active phases of the cell cycle except resting cells [41]. As a biomarker to estimate the proportion of dividing cells, we used it to analyze cell proliferation in xenografted testes. Cyclin-dependent kinase inhibitors (CDKIs) is one class of the cell cycle-regulating proteins, which could prevent transition from the G1 phase to S phase of the cell cycle [42]. CDKN1A and CDKN1B are two of the CDKIs and express in many cell types. Our results showed that the expression of MKI67 was significantly higher in the new integrative model group while CDKN1A and CDKN1B related to proliferation were not, indicating the active state in xenografted testes. DNA methylation is one of the epigenetic marks and plays a critically positive role in regulating testicular genes expression temporally and spatially during spermatogenesis in mammals, which is catalyzed by DNA methyltransferase (DNMT) enzymes. Two different mechanisms of DNA methylation have been identified: de novo and maintenance, which are catalyzed by DNMT1, DNMT3A and DNMT3B enzymes, respectively [43]. The lack of DNA methylation enzymes leads to different situations such as embryonic lethality, spermatogenic arrest, azoospermia, loss of imprinting [43]. The research showed that DNMT3B expression was low before birth, but the staining signals became relatively strong in spermatogonia and spermatocytes after birth [44]. Spermatogenesis is a dynamic, complex process, tightly regulated by the precise control of a variety of factors. DNA methylation and histone modification are closely related to each other in the process of gene expression, and successful regulation and control of gene expression requires close cooperation, and interaction of both of these mechanisms [45]. Moreover, specific domains of the DNMT3 proteins interact with histones, and this interaction is regulated by specific modifications of the amino terminal tails of histones [46]. It was reported that through exclusion of the DNA methyltransferase complex, methylated histone H3 lysine 4 (H3K4) protects associated DNA from DNA methylation [47], while H3K36me3 positively correlates with DNA methylation [48]. In this study, we found the expression of DNMT3B in the new model group was significantly higher while no significant difference in DNMT1 and DNMT3A, which needs further research on evaluating the function of DNMTs and the regulation of DNA methylation. And we considered the higher expression level of DNMT3B in xenografted testes was associated with oxidative damage after xenotransplantation.

In this study, we established a new integrative model and then evaluated the survival state of xenografted testes from morphological and functional perspective. The cultured testes could continue to develop in this new model, suggesting that it is a positive way to explore the EDCs effects on spermatogenesis after short-term and long-term exposure. The combination of testes organ culture and testes transplantation may provide a method for comparing EDCs’ short- and long-term effects after intervention in different species, especially for human because it is more acceptable on ethical issue.

## Conclusion

In this study, we established integrative model of immature neonatal testicular tissue, which consisted of organ culture in the first step and followed by xenotransplantation, and evaluated testicular development after organ culture and xenotransplantation. Our results revealed that the new integrative model could maintain the viability of testicular tissue and ensure the long-term survival in vivo with complete spermatogenesis. However, there were still some problems occurred in this model including altered testicular genes expression, formation of vacuoles and thin seminiferous epithelium, manifesting that oxidative damage may contribute to the testicular development lesion and needs further study in order to optimize this model. Moreover, it is necessary to isolate spermatids from xenografted testes and evaluate the fertilization ability. At the meantime, this new model might be a positive way to explore the EDCs effects on spermatogenesis in short term and long term.

## Data Availability

The datasets used and/or analyzed during the current study are available from the corresponding author on reasonable request.

## Abbreviations

SSCs: Spermatogonial stem cells
EDCs: Endocrine-disrupting chemicals
KSR: Knock-out serum replacement
PND: Postnatal day
AI: Apoptosis index
TEM: Transmission electron microscope
